# Pronounced mito-nuclear discordance and various *Wolbachia* infections in the water ringlet *Erebia pronoe* have resulted in a complex phylogeographic structure

**DOI:** 10.1038/s41598-022-08885-8

**Published:** 2022-03-25

**Authors:** Martin Wendt, Dustin Kulanek, Zoltan Varga, Laszlo Rákosy, Thomas Schmitt

**Affiliations:** 1 Senckenberg Deutsches Entomologisches Institut, Systematik und Biogeographie, Eberswalder Str. 90, 15374 Müncheberg, Germany; 2 Zoologisches Institut und Museum, Universität Greifswald, Loitzer Straße 26, 17489 Greifswald, Germany; 3 Department Evolutionary Zoology, Faculty of Science and Technology, University of Debrecen, Egyetem-tér 1, Debrecen, 4010 Hungary; 4 Department of Taxonomy and Ecology, Babes-Bolyai University, Str. Clinicilor 5–7, 3400 Cluj-Napoca, Romania; 5 Entomology, Zoology, Institute of Biology, Faculty of Natural Sciences I, Martin Luther University Halle-Wittenberg, 06099 Halle (Saale), Germany; 6 Entomology and Biogeography, Institute of Biochemistry and Biology, Faculty of Science, University of Potsdam, 14476 Potsdam, Germany

**Keywords:** Biogeography, Genetic markers, Infection, Entomology

## Abstract

Several morphological and mitochondrial lineages of the alpine ringlet butterfly species *Erebia pronoe* have been described, indicating a complex phylogenetic structure. However, the existing data were insufficient and allow neither a reconstruction of the biogeographic history, nor an assessment of the genetic lineages. Therefore, we analysed mitochondrial (COI, NDI) and nuclear (EF1α, RPS5) gene sequences and compared them with sequences from the sister species *Erebia melas*. Additionally, we combined this information with morphometric data of the male genitalia and the infection patterns with *Wolbachia* strains, based on a WSP analysis. We obtained a distinct phylogeographic structure within the *E. pronoe-melas* complex with eight well-distinguishable geographic groups, but also a remarkable mito-nuclear discordance. The mito-nuclear discordance in *E. melas* and *E. pronoe glottis* can be explained by different ages of *Wolbachia* infections with different *Wolbachia* strains, associated selective sweeps, and hybridisation inhibition. Additionally, we found indications for incipient speciation of *E. pronoe glottis* in the Pyrenees and a pronounced range dynamic within and among the other high mountain systems of Europe. Our results emphasize the importance of combined approaches in reconstructing biogeographic patterns and evaluating phylogeographic splits.

## Introduction

Butterflies are among the best-studied groups of invertebrates in Europe, but they nevertheless have great potential for harbouring previously overlooked biodiversity in the form of cryptic species^1,2^. However, unambiguous identification of cryptic species is difficult, due to similarities in morphology, behaviour, ecological niches, or individual genetic markers. Recent or rapid speciation events can promote cryptic diversity^3,4^, as can be seen in the genus *Erebia*. This genus has a particularly high diversification rate and is one of the most species-rich genera of European butterflies^5^. Numerous endemics, subspecies, diverse forms, and aberrations have been described^1,2,6,7^. The high intraspecific morphological variability combined with complex genetic structures has caused difficulties in distinguishing valid species^8^, and many other taxonomic and biogeographic questions have remained unanswered.

One of the unanswered questions relates to the status of the species *Erebia pronoe* (Esper, [1780]). *Erebia pronoe* is a European alpine butterfly and a character species of alpine rupicolous grasslands. There are several allopatric occurrences, due to the strong link to alpine habitats, among others in the Pyrenees, the Carpathians, and the Balkan mountain systems. Widely distributed species with allopatric distributions tend to have complex phylogeographic structures, from which difficulties in species delimitation may arise^9,10^. Thus, various morphological lineages, subspecies, and hybrid forms have been described for *E. pronoe* (e.g.^11–14^). Recently, three of these described lineages have been confirmed by genetic markers^1,7^. The mitochondrial gene segments analysed in these previous studies displayed multiple entities and possible indications of overlooked cryptic species within *E. pronoe*. Their results were based on haploid genetical markers, which are prone to influences like introgression, sex-specific behavioural differences^15^, and infection with parasitic bacteria such as *Wolbachia*^16,17^. Furthermore, important regions, such as the western Alps and the Balkan mountain systems, which probably served as dispersal corridors^7^, were not studied. A comprehensive understanding of the phylogeographic structure of this species was therefore lacking. A dense network of sampling sites across the range of the species, comparisons between biparental nuclear and maternally inherited mitochondrial genes, screening for *Wolbachia* infections, and a comparison with closely related taxa, like the sister species *Erebia melas*^10,18^, were needed to reconstruct the phylogeographic history of *E. pronoe*.

To obtain detailed information on phylogeographic patterns in this species, we sampled a dense network of sites across the entire range of *E. pronoe*: from the Pyrenees, through the entire Alpine region, and in the Carpathians, and the western and eastern Balkan mountain systems. We sequenced two nuclear and two mitochondrial gene segments and intensively screened all detected genetic lineages for infection with *Wolbachia* to address the problem of mito-nuclear discordance^19,20^. We performed a genetic comparison with the sister species *E. melas*, together with other representatives of the genus *Erebia* as outgroups, to put the phylogeographic analysis into a wider phylogenetic context. Additionally, we performed a morphometric analysis on the male genitalia to check for differences correlated with the genetically identified lineages.

We asked the following research questions:

1. Which of the morphologically described subspecies represent distinct genetic lineages?
2. Do mitochondrial and nuclear markers show similar phylogeographic patterns and how can possible differences be explained?
3. Where is the geographic origin of the species *E. pronoe* and what events led to the present distribution pattern with its phylogeographic structures?

## Results

### Genetic analyses

The combined sequences of COI and NDI (1181 bp) of the 124 specimens from the 27 populations had 46 haplotypes (genetic diversity parameters in Table 1). The most common haplotypes were H30 (39.2%), H17 (19.6%), H43 (19.6%), H22 (9.8%), H24 (9.8%), and H36 (9.8%). All other haplotypes had frequencies below 8%. The largest maximum p-distance between *E. pronoe* haplotypes was 0.0356 (Port de Larrau, F vs. Granchar, BG; Port de Larrau, F vs. Partnun, CH) with an overall average distance of 0.0179 (sd = 0.0127). The genetic distance between *E. pronoe* and its sister species *E. melas* ranged from p = 0.0094 (Königsstein, A; Loser, A) to 0.0376 (Port de Larrau, F); mean 0.0201 (sd = 0.0107). In both mtDNA markers, four non-silent mutations with amino acid change occurred in each Pyrenean population. Two changes in amino acid polarity occurred in each of the two mtDNA markers (see supplementary S1).

**Table 1:** Genetic diversity parameters of the mitochondrial and nuclear DNA markers of the separated and combined sequences of *Erebia pronoe*.

	COI	NDI	Combined mitochondrial DNA	EF1alpha	RPS5	Combined nuclear DNA
Nucleotid diversity Pi	0.01424	0.01332	0.01381	0.00333	0.00369	0.00346
Haplotyp diversity h	0.926	0.810	0.955	0.925	0.733	0.974
Segregation sites S	41	41	82	30	17	47
Average number of nucleotide differences k	8.90257	7.40401	16.30658	3.18257	2.13649	5.3191

**Figure 1:**
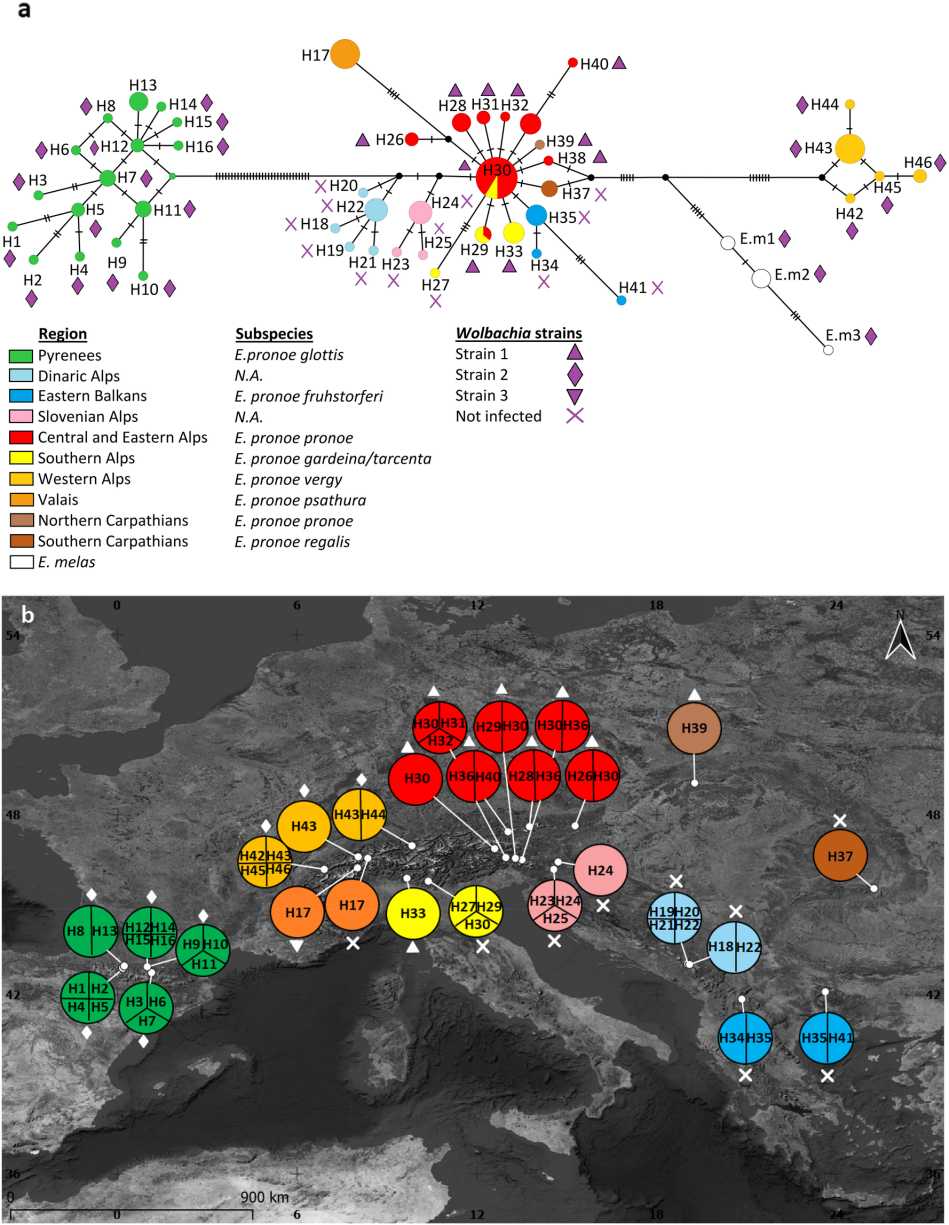
(**a**) TCS haplotype network based on the concatenated mtDNA haplotypes (COI, NDI) of *Erebia pronoe*. Mutational steps are shown by the number of hatch marks. The colour codes of each region and the corresponding subspecies are given in the legend (N.A. = no subspecies name available for this region). The reference haplotypes of *E. melas* are given in white. Detected *Wolbachia* infection strains are indicated by a symbol next to each haplotype. (**b**) Distribution of the identified concatenated mtDNA haplotypes (COI, NDI) among the populations of *E. pronoe*. The map was created with Qgis v.3.10.10 (Available online: http://qgis.osgeo.org).

The haplotype network based on both mtDNA markers distinguishes three main groups: a Pyrenean group, a western Alps group, and a group including all other regions (see Fig. 1). The Pyrenean group, which coincides with the occurrence of the subspecies *E. pronoe glottis* Fruhstorfer, [1920], has a mean genetical distance among populations ranging from 0.0014 to 0.0034. There is no dominant haplotype in this group, but systems with satellite haplotypes are emerging, especially around H12. The western Alps group, whose occurrence coincides with the subspecies *E. pronoe psathura* Fruhstorfer, [1920], has a markedly low genetic diversity (0.0002–0.0014) and possesses a dominant haplotype, i.e. H43. The third and by far most widespread group has a complex star-shaped structure centred on the dominant haplotype H30, which occurs in the central, eastern and southern Alps. Satellite haplotypes directly derived from it with only one mutation step have been found mainly in the central and eastern Alps, but also the southern Alps (H29, H33), the High Tatras (H39), and the Romanian Carpathians (H37). Two haplotypes with quite clear differentiation are also directly derived from H30, restricted to the Valais (H17) and the central Italian Alps (H27).

The western Balkan Peninsula system (i.e. Dinaric Alps) with the central haplotype H22 is genetically more distant from the eastern Alps (0.0034) than the system from the Slovenian Alps with the central haplotype: H24 (0.0021). The eastern Balkan Peninsula also has a similar system around H35, but is less differentiated from the dominant H30 (0.0016). The haplotypes of *E. melas* have similar genetic distances to the second (westalps 0.0115) and third groups (ostalps 0.0103) and are less differentiated from them than the Pyrenean group.

The 124 combined sequences of EF1**α** and RPS5 (1536 bp) resulted in 68 genotypes (genetic diversity parameters in Table 1). The most frequent genotypes were G64 (21.8%), G45 (6.5%), G31 (4.8%), G23 (4.8%). All other genotypes had a frequency of less than 3% (see supplementary S2 for the distribution). The maximum p-distance between *E. pronoe* genotypes was 0.004 (Passo San Marco, I versus Pyrenees; Granchar Rila, BG; Gletsch, CH). The average genetic distance was 0.0008 (sd = 0.00056). The genetic distance between *E. melas* and *E. pronoe* ranged from 0.0074 (Grindelwald, CH; Glockner Research Station, A; Alisnica, AL; Hochkönig, A) to 0.0094 (Passo San Marco, I); with an overall mean of 0.0077 (sd = 0.00035). There was no change of amino acids in the nuclear markers of the studied populations.

**Figure 2:**
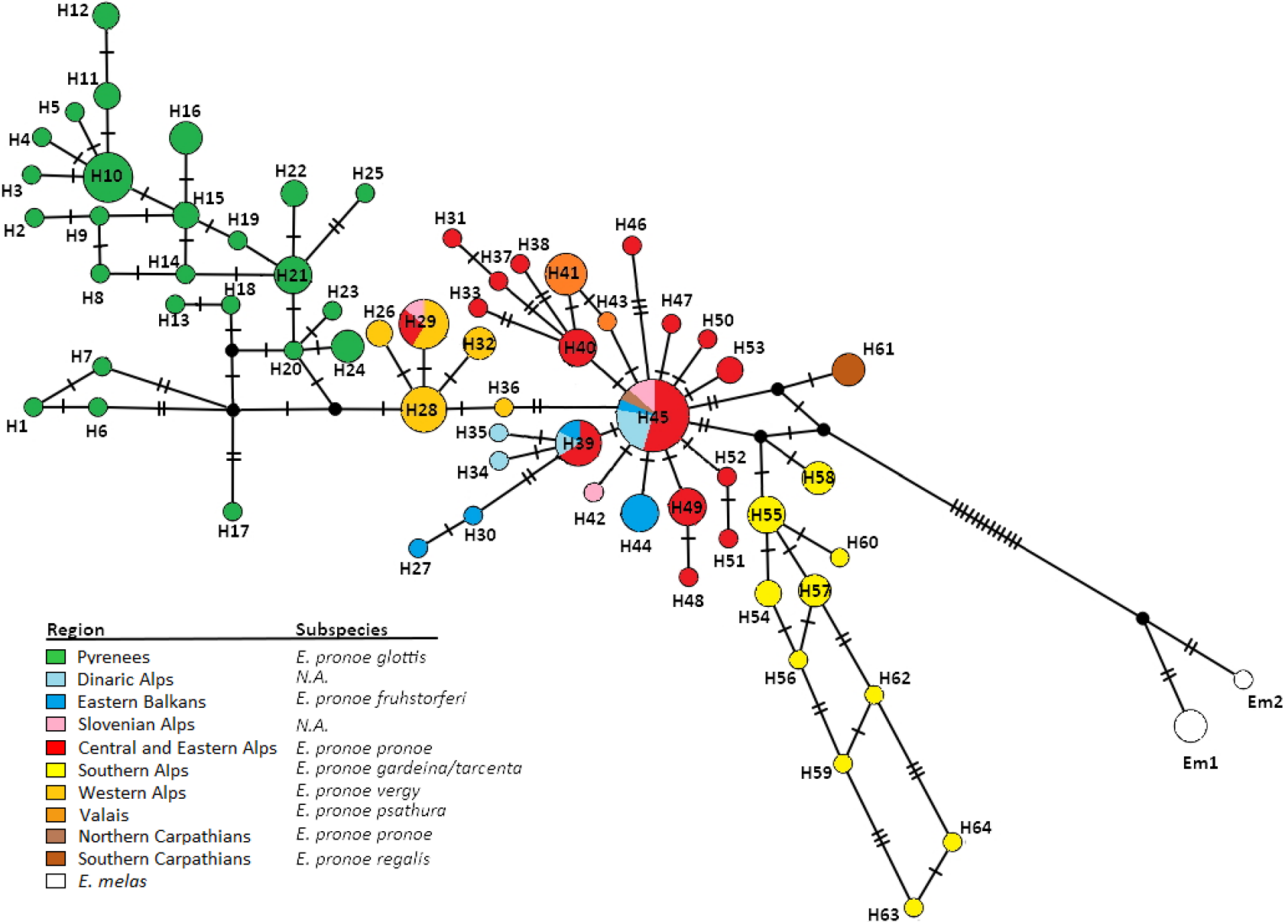
TCS haplotype network based on the concatenated phased nuclear DNA haplotypes (EF1α, RPS5) of *Erebia pronoe*. Mutational steps are shown by the number of hatch marks. The colour codes of each region and the corresponding subspecies are given in the legend (N.A. = no subspecies name available for this region). The reference haplotypes of *E. melas* are given in white. The geographical location of the haplotypes is given in the same colour scheme as in Fig. 1.

The haplotype network of the phased nuclear dataset was composed of four groups, three of which had complex structures (see Fig. 2). The Pyrenean group, the western Alps group, and the widespread group had internal structures similar to the mtDNA network. However, the populations from the central Italian Alps represented a separate fourth genetic group with high genetic diversity and were not part of the widespread group as they were in the mtDNA network. However, nuclear DNA did not yield clearly separated subgroups within the widespread group for the Slovenian Alps or the western and eastern Balkan mountain systems. The Romanian Carpathians were more differentiated from the central haplotype of the widespread group based on the nuclear genes, and less so based on mtDNA markers. The haplotype H29 of the western Alps group was rarely detected in the central, eastern and Slovenian Alps. Overall, differentiation between groups was significantly lower in the nuclear genes (compared to diversity and differentiation within groups) than in the mitochondrial genes. The western Alps group clustered between the Pyrenean group and the widespread group and was closest to the Pyrenean group. The separation between the sibling species *E. pronoe* and *E. melas* was significantly more prominent (12 vs. 18 mutational steps).

All examined specimens from the Pyrenean populations were infested with *Wolbachia* strain 2. This strain was also found in all western Alps populations. All except one of the Valais specimens were without *Wolbachia* infection. However, *Wolbachia* strain 3, which was exclusively found there, differed strongly from all other *Wolbachia* sequences (average p-distance 0.1766, see Table 2). *Wolbachia* strain 1 was detected in the central, eastern, and central Italian Alps, and in the High Tatras. No *Wolbachia* infections were detected in the populations of the Romanian Carpathians, Slovenian Alps, and Balkan Peninsula. The nominotypical *E. melas* population from the south-western Carpathians (Băile Herculane, RO) was infected with *Wolbachia* strain 2.

**Table 2:** Mean genetic distance of the three genetic strains of *Wolbachia* detected in *Erebia pronoe* based on the WSP gene fragment.

*Wolbachia* strain	*Wolbachia* 1	*Wolbachia* 2
*Wolbachia* 1		
*Wolbachia* 2	0.0393	
*Wolbachia* 3	0.1682	0.185

**Figure 3:**
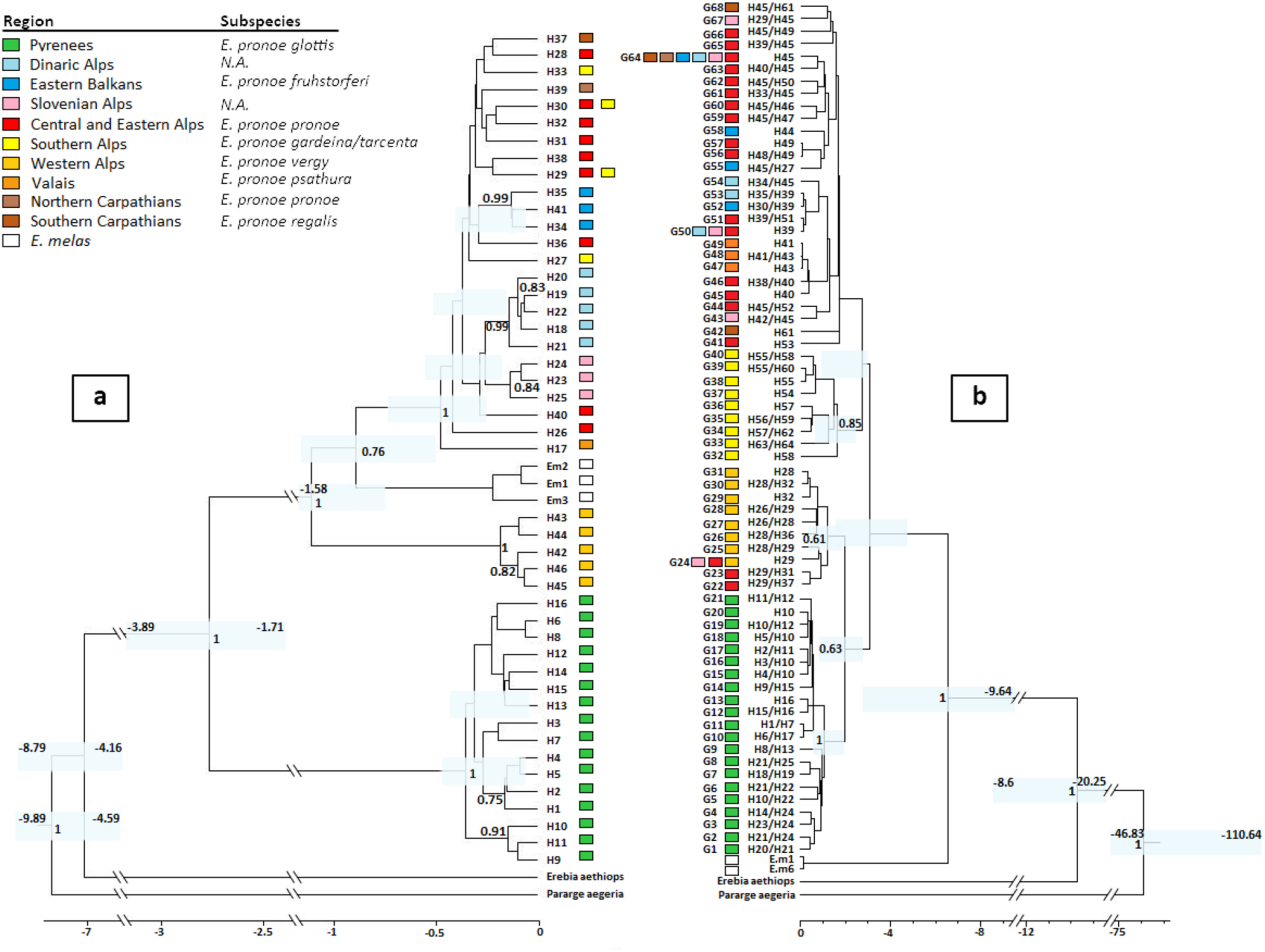
Bayesian phylogeny based on concatenated (**a**) mtDNA haplotypes (COI, NDI) and (**b**) nuclear haplotypes (EF1α, RPS5) of *Erebia pronoe*. Numbers next to the nodes: Bayesian posterior probabilities > 0.7; light blue node bars: 95% highest posterior density of node ages (age is explicitly given as number if bar includes a cut in the timeline). The colour codes of each region and the corresponding subspecies are given in the legend (N.A. = no subspecies name available for this region). The reference haplotypes of *E. melas* are given in white. The geographical location of the haplotypes is given in the same colours as in Fig. 1.

Model testing revealed four partitions in the mtDNA dataset: HKY + I (Gen1:1, Gen2:3), K80 + I (Gen1:2), F81 (Gen1:3), and HKY + I (Gen2:1, Gen2:2). Because K80 + I and F81 are submodels of the HKY model and cannot be selected separately, we used the HKY model instead. Model testing resulted in three partitions for the nuclear dataset: JC (Gen1:1, Gen1:3, Gen2:2), K80 + I (Gen1:2, Gen2:1), F81 (Gen2:3). The partitions suggested by the model test did not result in a successful run with stable ESS values for the nuclear data set. Therefore, several test runs were performed and the coalescent constant population size tree model with HKY substitution model gave the best results (see “Material and methods”; supplementary S3). Both trees in the Beast analysis identified the Pyrenees and western Alps as distinct groups (see Fig. 3). The Slovenian Alps, the western and the eastern Balkan mountain systems groups are separated in the mtDNA tree but are included in the widespread group in the nuclear tree. Conversely, the central Italian southern Alps are part of the widespread group in the mtDNA tree but occupy a clearly separated position in the nuclear tree. We found the strongest discordance between the two analyses and the nuclear and mitochondrial markers in the position of *E. melas*. In the mitochondrial markers, *E. melas* is nested within *E. pronoe*, whereas *E. melas* is the sister to *E. pronoe* in the nuclear tree. Analysis of mismatch distributions of pairwise sequence differences revealed a bimodal distribution in the mitochondrial DNA and a geometric distribution in the nuclear dataset (see supplementary S4, S5). RASP model test reported the Dispersion-Extinction-Cladogenesis (DEC + J) as the best model for condensed trees based on AICc and LnL criteria. RASP analyses with DEC + J setting failed to separate the region of origin for either data sets, displaying every region as equally likely. In contrast, the Bayesian inference for discrete areas (BayArea) approach indicated the eastern Alpine region (region "c") as the most likely area of origin for the nuclear markers and the Carpathian region (region “e” for the mitochondrial markers (see supplementary S6–S9).

### Genital morphology

The principal component analysis showed that the first two principal components explained most of the variation (48.6% and 21.0%, see supplementary S10–S12). According to these components, the valves of *E. pronoe* and *E. melas* were distinguishable from each other, whereas an intraspecific separation within *E. pronoe* was not possible. K means clustering for k = 3 (see supplementary S13) resulted in two clusters with all *E. pronoe* lineages mixed and a third cluster with all *E. melas* specimens. Landmarks 8–10 had the highest explanatory power of the 45 landmarks (see supplementary S14).

## Discussion

*Erebia pronoe* exhibits highly structured and strongly differentiated mitochondrial lineages, which are consistent with the distribution of previously described morphotaxa and analyses of Dincă et al.^10^ These genetic lineages are also reflected to varying degrees in the nuclear markers. The observed mito-nuclear discordances can be explained by different evolutionary rates of genetic markers, the effects of *Wolbachia* infections, and introgression. These aspects are discussed in more detail in the following sections on the phylogeographic history of this species complex.

### Mito-nuclear discordance and the systematic status of *Erebia melas*

Based on genital morphology and nuclear markers, *E. melas* represents a distinct group to *E. pronoe*. The common area of origin of both species was probably located in the eastern Alps, which is supported by a RASP analysis based on the nuclear markers. However, *E. melas* acts as an ingroup of *E. pronoe* based on the mitochondrial markers, and a RASP analysis indicates a common origin for both taxa in the Carpathian region. Since most *Erebia* species in Europe have at least parts of their distribution in the Alps^21^ and are adapted to Alpine environments and habitats^22,23^, we consider an eastern Alpine origin of the ancestor of *E. pronoe* and *E. melas* more likely. This hypothesis subsumes the assumption that the genetic proximity on the mitochondrial level was probably caused by hybridisation and introgression events, which could have occurred as a result of several eastward advances of *E. pronoe* to the Balkan Peninsula (see below). This seems plausible, because the ability and tendency of *E. pronoe* to hybridise with other *Erebia* species have been demonstrated repeatedly^12,24,25^.

The existence of *Wolbachia* strain 2 in both species, and its distribution from the Pyrenees (in *E. pronoe*) to the Balkan Peninsula (in *E. melas*) also speaks for a common origin of both species. Thus, *Wolbachia* strain 2 might represent the ancient strain present in the common ancestor of this species group, surviving today at the geographic margins (i.e. Pyrenees, western Alps, Balkan Peninsula), but which at some time was replaced in the centre of the butterfly’s range (i.e. the eastern and central Alps) by strain 1. The link between co-occurrence in a common area and prevalence of one *Wolbachia* strain was also recently demonstrated in other *Erebia* species^26^ and might facilitate mitochondrial introgression^27^.

### Intraspecific differentiation and glacial refugia of *Erebia pronoe*

The Pyrenean region is inhabited by one of the oldest and most differentiated intraspecific lineages of *E. pronoe*. The high genetic diversity in the Pyrenees speaks for large effective population sizes throughout time, enabled by mostly altitudinal shifts in response to climatic cycles, and a lack of major genetic bottlenecks. Compared to the Pyrenean group, the genetic diversity of the western Alpine populations, also well differentiated from all other groups, is lower. This lower diversity was probably the result of repeated cold stage retreat to a geographically more restricted refugium at the foot of the south-western Alps, a well-known refugial area for numerous species^28^.

We cannot say conclusively whether the populations in the Pyrenean region or in the western Alps differentiated first, due to the contradictory genetical markers. The higher evolutionary rate of the mitochondrial markers, the allopatric distribution, and the hybridisation with diverse *Erebia* species may have led to a greater differentiation of the Pyrenees and/or a loss of the genetic link between the western Alps and the Pyrenees. Since a link between the western Alps and the Pyrenees is still well reflected in the nuclear data set and by the shared *Wolbachia* strain 2, we consider the most likely scenario to be an early Pleistocene or even Pliocene expansion from the western Alps to the Pyrenees, with subsequent isolation and differentiation. Thus, the Pyrenees-western Alps populations might first have separated as one group from an eastern Alps group s.l., as suggested by nuclear information, and not in two independent events, as suggested by mitochondrial genes.

Simultaneously to the split between western Alps and Pyrenees, a separation of the eastern Alpine group s.l. into a southern Alpine subgroup and an eastern Alpine subgroup should have occurred. The southern Alpine subgroup displays a high genetic diversity in their nuclear markers, but a significantly lower diversity in the mtDNA. This might be explained by the existence of a cold-stage refugial area in the southern Alps or their margin, supporting the constant survival of large populations, but also a reshaping of the mtDNA patterns through introgression from the eastern Alpine subgroup during secondary contact when both subgroups expanded into formerly glaciated east-central Alpine areas. The isolated occurrence of *Wolbachia* strain 1 and mitochondrial haplotypes H29 and H30 (shared with the eastern Alps subgroup) in the southern Alps further support the hypothesis of gene flow from the eastern Alpine region into the southern Alpine populations and vice versa.

The eastern Alpine subgroup probably survived glacial periods in a large, cohesive refugium at the eastern edge of the Alps, as has been demonstrated for numerous other species^28^. This area is also seen as a potential centre of origin of the entire taxon. From there, a recent (most likely postglacial) dispersal must have taken place, which should be responsible at least partly for the star-like pattern of this group in both mitochondrial and nuclear haplotype networks. However, further dispersal events out of the eastern Alps during previous interglacials and maybe even going back to the Pliocene have to be postulated to explain the entire range dynamics in *E. pronoe*.

Apparently, multiple advances out of the eastern Alps into the Balkan mountain systems have taken place from several independent glacial refugia in the region, as indicated by the different mtDNA lineages in Slovenia, western Balkan mountains, and eastern Balkan mountains. A separation between the eastern and western Balkans, and hence also separate glacial refugia in both areas, was frequently observed for mountain taxa^28,31^. This pattern may have resulted from a succession of independent dispersal events from the eastern Alps throughout the younger Pleistocene, with subsequent regional extinction events and/or independent dispersal events across the Carpathians, as has been demonstrated for numerous other species^29^.

A similar pattern of two independent colonisation events also applies to the Carpathians. Thus, the highly isolated populations in the south-eastern Carpathians must go back to an older expansion out of the eastern Alps. This probably took place during one of the last interglacial phases. The route most likely followed the Carpathian arc, but only a few populations survived at their south-eastern edge. This underlines the phylogeographic independence of this part of the Romanian Carpathians, which is also supported by studies on numerous other mountain species^30–32^. On the other hand, the Tatra mountains, as the northernmost part of the Carpathians, were colonised very recently, most likely postglacially, out of the eastern Alpine area. The strong and rather recent link between these two areas is also supported by phylogeographic studies on many taxa^30,33,34^.

Because of the slower evolutionary rate of nuclear DNA and the resulting incomplete lineage sorting, nuclear markers can contribute little to the reconstruction of these presumably recent events. In line with that, the Valais lineage also has little nuclear differentiation but is clearly distinguished from the western and eastern Alpine lineages by the exclusive mtDNA haplotype H17 and *Wolbachia* strain 3. The presence of a single, highly differentiated mtDNA haplotype and an exclusive *Wolbachia* strain indicates a selective sweep. This lineage most likely represents a chronological relict of an interglacial expansion of the eastern Alpine subgroup to the western-central Alps surviving since then in this area, finding glacial refugia in nearby unglaciated areas and becoming infested by a *Wolbachia* strain not present in any other *E. pronoe* lineage, hence accelerating its differentiation.

Another selective sweep was probably the cause of the mito-nuclear unconformity in the southern Alps lineage. The occurrence of the mtDNA haplotypes H29 and H30 and the *Wolbachia* strain 1 indicate mitochondrial hybridisation between the eastern and southern Alpine lineages during an expansive interglacial phase. As a result, *Wolbachia* infection probably occurred, which might have impoverished the mitochondrial diversity of the southern Alps lineage.

### Consequences for subspecific differentiation in *Erebia pronoe*

In general, the support given by our data for the so-far described subspecies decreases from west to east. *Erebia pronoe glottis* Fruhstorfer, 1920, distributed in the Pyrenees, represents the best-supported subspecies. Fixed mitochondrial amino acid changes emphasize the distinctness of this taxon, which might be well advanced in the process of speciation; we cannot even exclude the possibility that it has already reached full species rank. The genetic separation of the western Alps from the Valais, geographically separated along the main Alpine ridge, justifies the recognition of the taxa *E. pronoe vergy* (Ochsenheimer, 1807) and *E. pronoe psathura* Fruhstorfer, 1920, respectively, and is supported by both marker sets as well as by the existence of two different *Wolbachia* strains. The eastern Alpine subgroup resembles the nominotypical *E. pronoe pronoe*. The existence of at least one lineage in the southern Alpine area is supported by both marker sets. A finer separation based on the mitochondrial markers is not possible, because of recent introgression events affecting east Alpine haplotypes, as also indicated by the existence of *Wolbachia* strain 1. This population group could be assigned to the taxon *E. pronoe gardeina* Schawerda, 1924, or to *E. pronoe tarcenta* Fruhstorfer, 1920, considering their ranges. Nevertheless, a final decision requires further regional studies. *Erebia pronoe fruhstorferi* Warren, 1933 was accepted to be widely distributed in the Balkan mountain systems. However, our data suggest independent lineages in the western and eastern Balkan mountain systems of which only the eastern populations can be assigned to this taxon. The lineage of the Slovenian Alps is primarily based on mitochondrial markers and morphological characteristics^7^. The existence of an independent lineage for the highly isolated populations in the southern Carpathians, justifies the subspecies status of *E. pronoe regalis* Hormuzachi, 1937. Both marker sets display a differentiation, which was more pronounced in the nuclear than in the mitochondrial DNA.

## Material and methods

### Study species

The water ringlet butterfly *Erebia pronoe* (Esper 1780) is a typical species of gravel-interspersed rough grasslands and also wet meadows of the high montane to alpine zone^35^. *Erebia pronoe* is widespread from the Cordillera Cantabrica through the Pyrenees and into the Alps. However, the species occurs only as scattered and isolated populations in the Carpathians and the Balkan mountain systems*. Erebia pronoe* is a univoltine species with a flight period from late July to mid-September and overwinters as a L1 larva. The larvae feed on *Festuca ovina* and *F. quadriflora*^22^.

### Sampling design

124 specimens representing 27 populations (1–8 specimens, mean: 4.6 specimens, see Fig. 1b; supplementary S15) were collected from the Pyrenees to the Tatra Mountains and the Balkan Peninsula during the summers 2000–2014. Butterflies were captured with a hand net and frozen in liquid nitrogen in the field. The specimens were stored in a freezer at − 80 °C until analysis.

### Genetic analysis

#### DNA sequencing.

Genomic DNA was extracted from one leg using the EZNA Tissue DNA Kit (Omega Bio-Tek, Norcross, USA). The manufacturer's protocol was followed.

Variation in two mitochondrial genes and two nuclear genes was examined for the entire dataset. We sequenced the barcoding region encoding a fragment of cytochrome c oxidase subunit I (COI, 658 bp) and NADH dehydrogenase subunit I (NDI, 554 bp). COI was amplified with the primer pair LEP-F1 (5′-ATTCAACCAATCATAAAGATATTGG-3′) and LEP-R1 (5′-TAAACTTCTGGATGTCCAAAAAATCA-3′)^36^ using the following PCR protocol: 95 °C for 5 min, followed by 38 cycles at 95 °C for 30 s, 49 °C for 90 s, 72 °C for 60 s, and terminated with a final extension step at 68 °C for 30 min. NDI was amplified using the primer pair FAW-NDI (5′-TTCAAACCGGTGTAAGCCAGG-3′) and FAW-16S (5′-TAGAATTAGAAGATCAACCAGC-3′)^37^ and the following PCR protocol: 95 °C for 5 min, 33 cycles at 95 °C for 30 s, 56 °C for 90 s, 72 °C for 60 s, and terminated at 68 °C for 30 min. Furthermore, the eukaryotic translation elongation factor 1 alpha (EF1α, 957 bp) and ribosomal protein S5 (RPS5, 610 bp) were sequenced. The nuclear markers isocitrate dehydrogenase (IDH, 709 bp), Nedd2-like caspase (NC, 610 bp), glyceraldehyde-3-phosphate dehydrogenase (GAPDH, 663 bp), and wingless (WG, 400 bp) did not provide sufficient variability among the Pyrenees, the western, southern, and eastern Alps. Therefore, only a test series of each region was examined and no further analysis of these markers was performed. For PCR protocols and primers, see^38,39^.

The *Wolbachia* surface protein-coding gene (WSP, 549 bp) was amplified with the primer pair wsp81F (5′-TGG TCC AAT AAG TGA TGAAGA AAC-3′) and wsp 691R (5′-AAA AAT TAA ACG CTA CTC CA-3′)^40^ using the following PCR protocol: 95 °C for 5 min, followed by 40 cycles at 95 °C for 30 s, 54 °C for 90 s, 72 °C for 60 s, and terminated with a final extension step at 68 °C for 30 min.

The PCR products were loaded onto a 1.4% agarose gel and stained with GelRed (Biotium, Fremton, USA) to check for successful amplification. Primers and dNTPs were deactivated with a mixture of FastAP and exonuclease I (Thermo Scientific, Dreieich, Germany). The amplified products were shipped to Macrogen Europe (Amsterdam, The Netherlands). Both sense and antisense strands were sequenced.

#### Data analyses

Our mitochondrial and nuclear sequences were assembled using GENEIOUS v. 10.2.3 and aligned using CLUSTALW implemented in BIOEDIT v. 7.2.6.1. The sequences of each marker set were concatenated and checked for stop codons using GENEIOUS. The GenBank accession numbers are given in the data availability statement and in more detail with the corresponding haplotypes/genotypes in supplementary S16. The nuclear dataset was phased in DNASP with default settings. Haplotype frequency, haplotype diversity (h), number of segregating sites (S), nucleotide diversity per gene (pi), and average number of nucleotide differences (k) were calculated using DNASP v. 6. The combined dataset was used to construct a TCS haplotype network with default settings using POPART v. 1.7. A mismatch analysis was performed separately for both marker sets in R using the packages ‘adegenet’ and ‘pegas’.

A Bayesian tree was reconstructed based on the mitochondrial dataset and based on the nuclear dataset using BEAST v. 2.5. Published data for *Pararge aegeria* and *Erebia aethiops* were used as outgroups. The partition and substitution models were estimated using PARTITIONFINDER v. 2.1.1 based on the lowest Akaike Information Criterion (AIC). For the mtDNA dataset, the HKY model with empirical base frequencies and a gamma distribution with a category number of 4 were selected. We performed several analyses testing every option offered by Beast to select the best-fitting tree model. Based on the logarithmic likelihood and the explained sum of square (ESS) values, the coalescent constant population model performed best and was therefore selected for the final analysis. We used a relaxed clock log normal setting for the molecular clock and a clock rate of 0.0177^41^.

For the nuclear dataset, using the settings proposed by Partitionfinder led to the early termination of the analysis. We obtained stable analyses and parameter values with ESS above 200, by avoiding partitions and using the HKY model with empirical base frequencies and a gamma distribution with a category number of 4. Based on the logarithmic likelihood and the explained sum of square (ESS) values, the coalescent exponential population model performed best and was therefore selected for the final analysis. We used a relaxed clock log normal setting for the molecular clock and a clock rate of 0.00177^41^.

We performed the analyses with 40 million generations, collecting every 4000 iterations. After checking the MCMC chain for convergence in TRACER v. 1.7.1, a burn-in of 10% was applied. Three individual runs were performed and combined with LOGCOMBINER v. 1.8.4. TREEANNOTATOR v. 2.5 was used to generate a summary tree with common ancestral probability. FIGTREE v. 1.4.4 was used for the visualization.

The same protocol was followed to obtain an input and consensus tree for both datasets for an ancestral occurrence reconstruction analysis using RASP v. 4.2. This analysis was performed for both marker sets of *E. pronoe* and the outgroups of *E. melas*, *E. aethiops*, and *P. aegeria*. The individual sequences were assigned to occurrences coded as consecutive letters (A: Pyrenees; B: western Alps; C: eastern Alps; D: Balkan mountain systems; E: Carpathians). Based on the results of the BioGeoBears model test, an S-DEC analysis was performed with the maxarea = 5 setting, as well as a Bayesian Binary MCMC with 50,000 cycles and K81 instead of JC.

### Preparation of genitalia

The genital capsule was removed with dissecting needles and macerated in a 10% potassium hydroxide solution for at least twelve hours. The genital apparatus was cleaned and the valvae and aedeagus were separated from the tegumen. The specimens were then fixed in euparal or Canada balsam on a microscope slide. Images were captured using a camera (Leica CFV450) mounted on a binocular microscope. To combine images of different depths of focus, we used a focus stacking technique (Helicon Focus). A combination of 6 landmarks and 39 sliding semi-landmarks^42^ was used with the help of TPS software^43–45^. A general Procrustes analysis was performed to obtain relative warps. The calculation of K Means clustering, hierarchical clustering based on Euclidian distance, and the generation of graphs were performed using R version 3.3.2^46^, the implemented package R stats, and the R package networkD3 0.4^47^.

## Supplementary Information

Supplementary Information.

## Data Availability

The datasets used and/or analysed during the current study are available from the corresponding author on reasonable request. All data generated or analysed during this study are included in this published article [and its supplementary information files]. GenBank accession number MZ190632-MZ190677 (*E.p* CO1); MZ345012-MZ345057 (*E.p* ND1); MZ190563-MZ190631 (*E.p* RPS5); MZ190749-MZ190750 (*E.m* RPS5); MZ190678-MZ190746 (*E.p* Ef1alpha); MZ190747-MZ190748 (*E.m* Ef1alpha), MZ358189-MZ358191 (WSP); MZ358183- MZ358185 (*E.m* CO1); MZ358186-MZ358189 (*E.m.* ND1).
