# Supplementary material for: Pronounced mito-nuclear discordance and various Wolbachia infections in the water ringlet Erebia pronoe have resulted in a complex phylogeographic structure
Source: Sci Rep. 2022 Mar 25;12:5175. doi: 10.1038/s41598-022-08885-8 (PMC8956704; doi:10.1038/s41598-022-08885-8)
Supplement: Supplementary file 1 — Supplementary Information. [file 41598_2022_8885_MOESM1_ESM.doc]

Pronounced mito-nuclear discordance and various *Wolbachia* infections in the water ringlet *Erebia pronoe* have resulted in a complex phylogeographic structure

Martin Wendt[[1]](#footnote-2)*, Dustin Kulanek^1^,[[2]](#footnote-3), Zoltan Varga[[3]](#footnote-4), Laszlo Rákosy[[4]](#footnote-5)**,** Thomas Schmitt^1,5,6^

S1 Non-silent mutation in the mtDNA marker of *E. pronoe* in the Pyrenees populations with details of the base pairs.

|  | CO1 | ND1 |
| --- | --- | --- |
|  | 27 AA.: Asparagin(ASN)->Threonin(THR) | 6 AA.:Threonin(THR)->Methionin(Met)  (polar->unpolar) |
|  | 29 AA.: Serin(SER)->Glycin(Gly)  (polar-unpolar) | 13 AA.: Glycin(Gly)-> Valin(Val) |
|  | 30 AA.: Phenylalanin(Phe)->Serin(Ser) (unpolar ->polar) | 68 AA.: Methionin(Met)-> Valin(Val) |
|  | 67 AA.: Valin(Val)->Isoleucin(Ile) | 122 AA.: Methionin(Met)->Threonin(THR)  (unpolar->polar) |


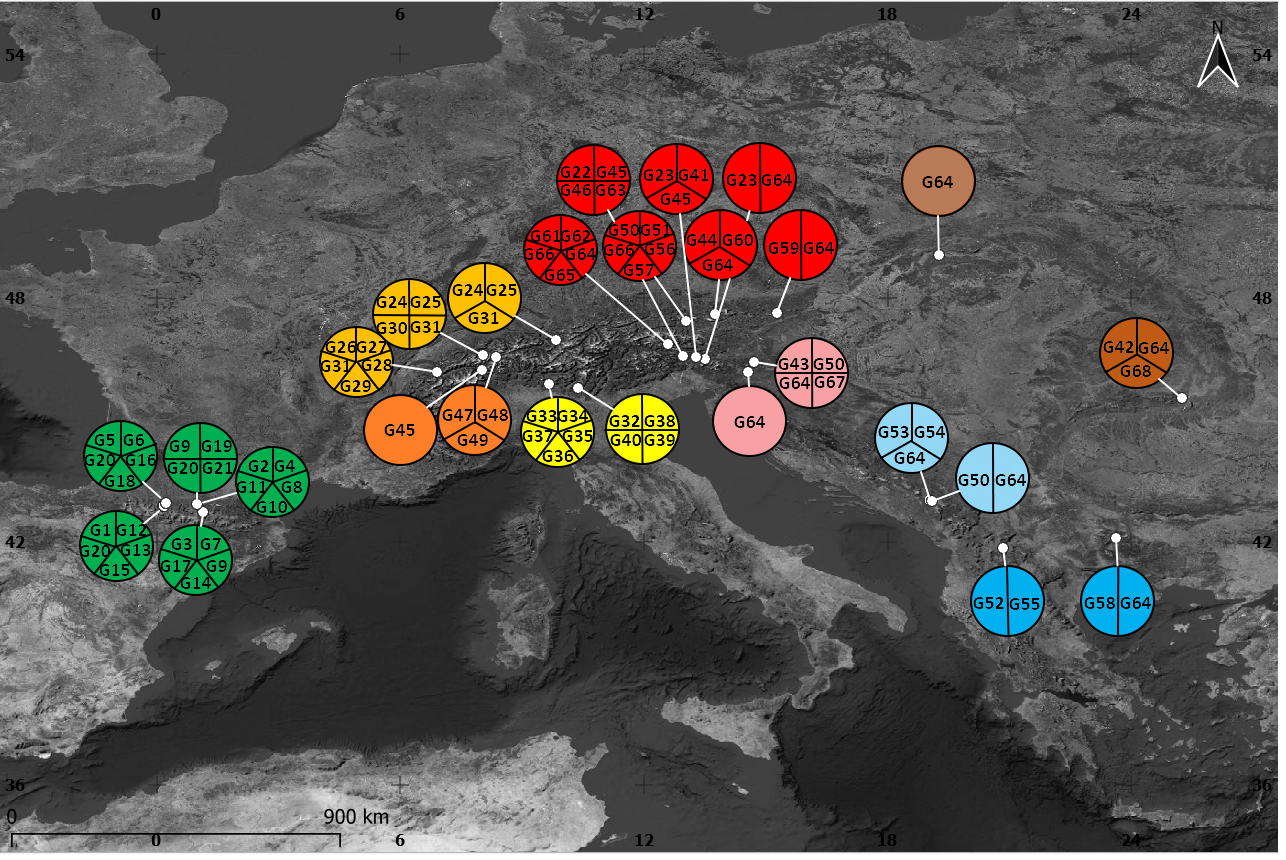


**S 2 Distribution of the identified concatenated nuclear DNA haplotypes (Ef1α, RPS5) haplotypes among the populations of *E. pronoe*. The map was created with**

Qgis v.3.10.10 (Available online: http://qgis.osgeo.org).

S 3 Tested Beast tree models with respective posterior, likelihood prior values and the number of ESS values below 200 for the nuclear data set of *E. pronoe*.

|  | Posterior | Likelihood | Prior | ESS < 200 |
| --- | --- | --- | --- | --- |
| Yule model (with partition) | 45672.036 | -2511.205 | 48183.241 | 28 |
| Calibrated Yule model (with partition) | - | - | - | - |
| Birth Death model (with partition) | **-1191.461** | **-2910.297** | **1718.836** | 10 |
| Coalescent constant population model (with partition) | **88973.685** | **-2917.832** | **9189.517** | 31 |
| Coalescent exponential population model (with partition) | **-49485.186** | **-2480.265** | **-51965.451** | 24 |
| Yule model | **-3386.803** | **– 3277.856** | **-108.948** | 5 |
| Calibrated Yule model | - | - | - | - |
| Birth Death model | **-3342.947** | **– 3296.598** | **-46.35** | 5 |
| Coalescent constant population model | **-3528.819** | **– 3293.915** | **-234.904** | - |
| Coalescent exponential population model | **-3520.63** | **-3293.249** | **-227.381** | - |


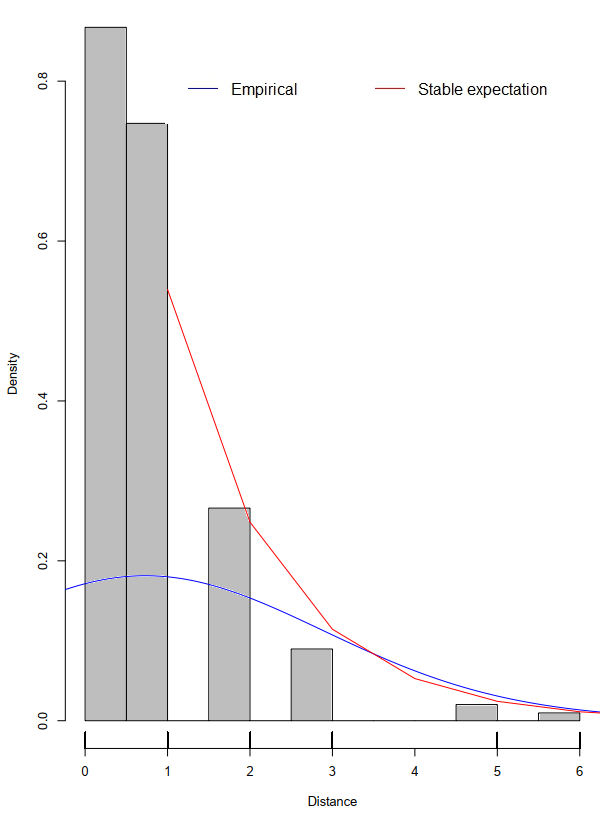


*.*


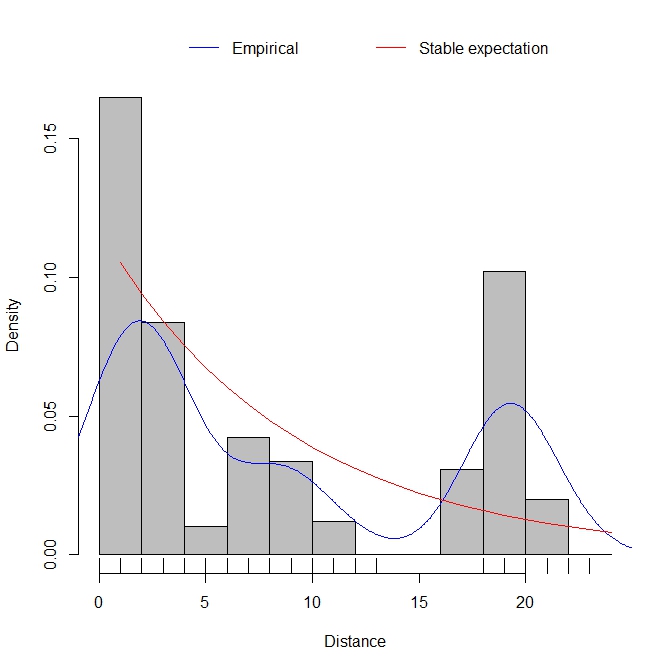


**S4 Mismatch distribution analysis of the mtDNA markers of *E. pronoe* generated in R using the packages "adegenet" and "pegas".**

**S5 Mismatch distribution analysis of the nuclear DNA markers of *E. pronoe* generated in R using the packages "adegenet" and "pegas".**


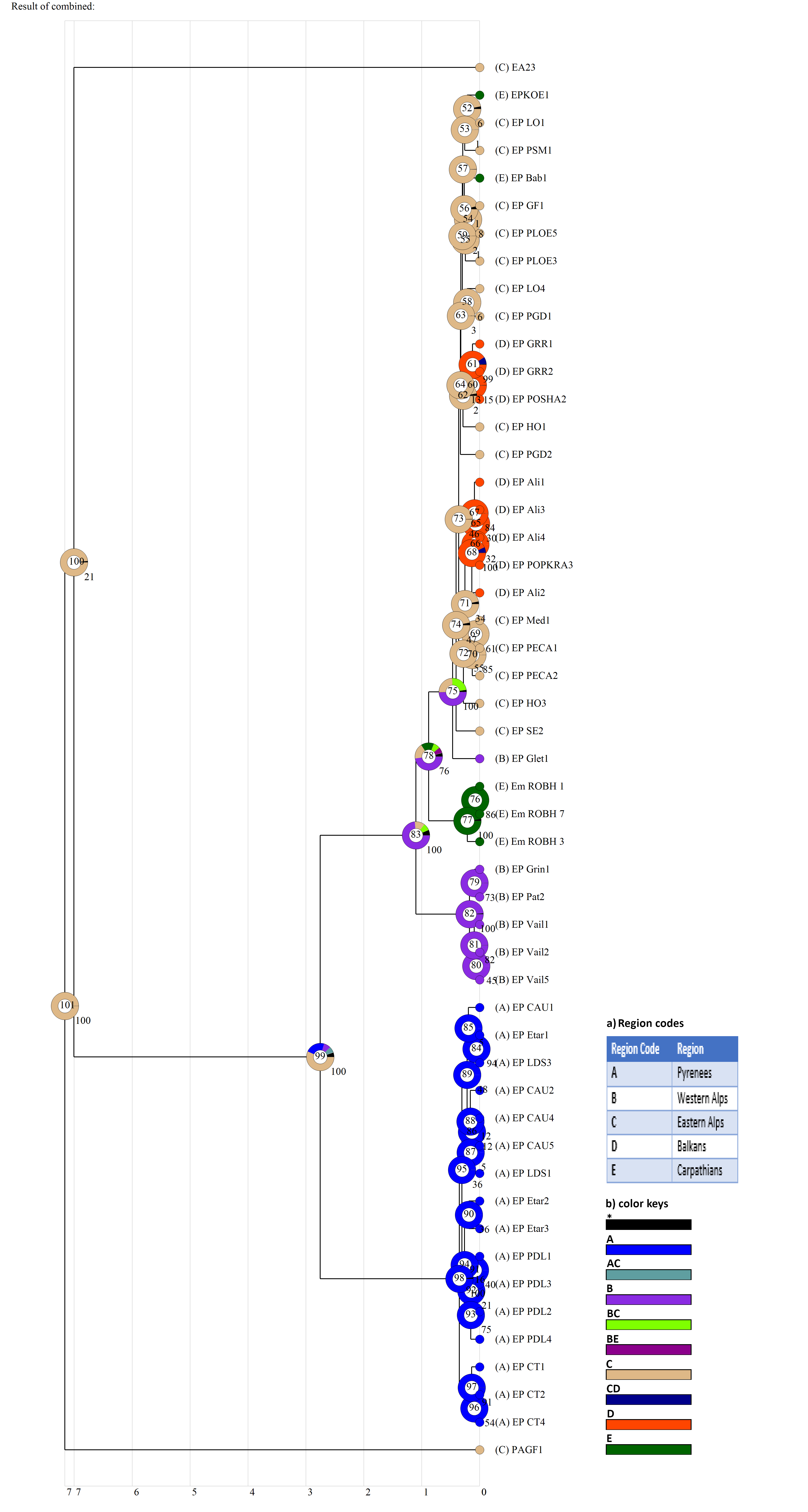


S6 Graphical output from Bayesian analysis (exported from RASP). Graphical results of ancestral distributions at each node of the *E. pronoe* group obtained by Bayesian Binary MCMC analysis based on the mtDNA dataset. Pie charts at each node show probabilities of alternative ancestral ranges with the posterior probability for the node next to it. a) Region codes; b) Color key to possible ancestral ranges at different nodes; black with an asterisk represents other ancestral ranges. Tip labels contain the area codes and correspond to the GenBank accessions codes of S15.

S7 Results of the RASP Model Test for the mtDNA dataset.

|  | LnL | numparams | d | e | j | AICc | AICc_wt |
| --- | --- | --- | --- | --- | --- | --- | --- |
| DEC | -43.77 | 2 | 0.1 | 0.14 | 0 | 91.81 | 4.80E-06 |
| DEC+J | -31.85 | 3 | 1.00E-12 | 2.90E-08 | 0.016 | 70.24 | 0.23 |
| DIVALIKE | -40.2 | 2 | 0.11 | 4.80E-09 | 0 | 84.67 | 0.0002 |
| DIVALIKE+J | -30.65 | 3 | 1.00E-12 | 1.00E-12 | 0.017 | 67.82 | 0.77 |
| BAYAREALIKE | -51.1 | 2 | 0.15 | 0.85 | 0 | 106.5 | 3.10E-09 |
| BAYAREALIKE+J | -36.73 | 3 | 1.00E-07 | 0.23 | 0.022 | 80 | 0.0017 |


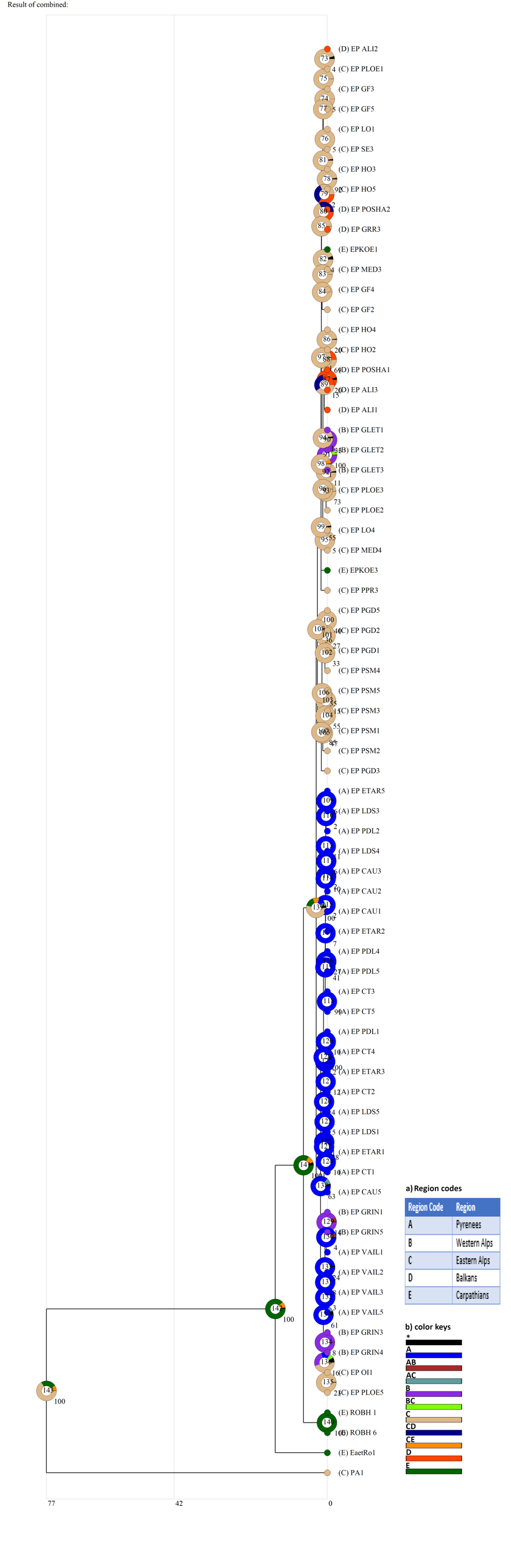


S8 Graphical output from Bayesian analysis (exported from RASP). Graphical results of ancestral distributions at each node of the *E. pronoe* group obtained by Bayesian Binary MCMC analysis based on the nuclear DNA dataset. Pie charts at each node show probabilities of alternative ancestral ranges with the posterior probability for the node next to it. a) Region codes; b) Color key to possible ancestral ranges at different nodes; black with an asterisk represents other ancestral ranges. Tip labels contain the area codes and correspond to the GenBank accessions codes of S15.

**S9 Results of the RASP Model Test for the nuclear DNA dataset.**

|  | LnL | numparams | d | e | j | AICc | AICc_wt |
| --- | --- | --- | --- | --- | --- | --- | --- |
| DEC | -82.3 | 2 | 0.027 | 0.037 | 0 | 168.8 | 3.60E-10 |
| DEC+J | -60.22 | 3 | 1.00E-12 | 1.00E-12 | 0.03 | 126.8 | 0.47 |
| DIVALIKE | -84.49 | 2 | 0.039 | 0.05 | 0 | 173.1 | 4.00E-11 |
| DIVALIKE+J | -60.74 | 3 | 1.00E-12 | 1.00E-12 | 0.031 | 127.8 | 0.28 |
| BAYAREALIKE | -113.5 | 2 | 0.045 | 0.11 | 0 | 231.1 | 1.10E-23 |
| BAYAREALIKE+J | -60.84 | 3 | 1.00E-07 | 1.00E-07 | 0.031 | 128 | 0.25 |


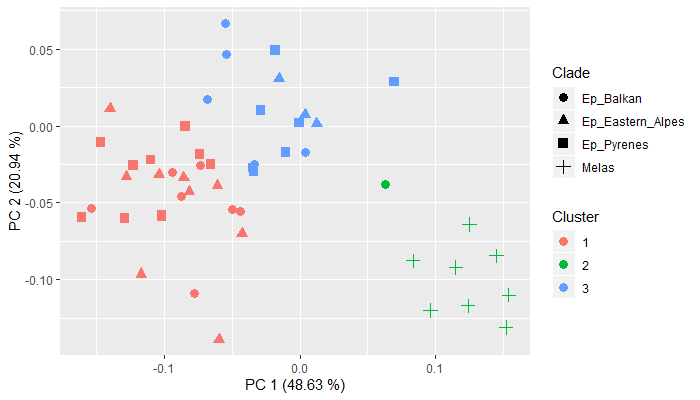


S10 K means Clustering (K = 3) (49 landmarks) of penis valves of E. pronoe and E.melas as reference species. First principal component (PC1) is on the x-axis, second principal component(PC2) on the y-axis.


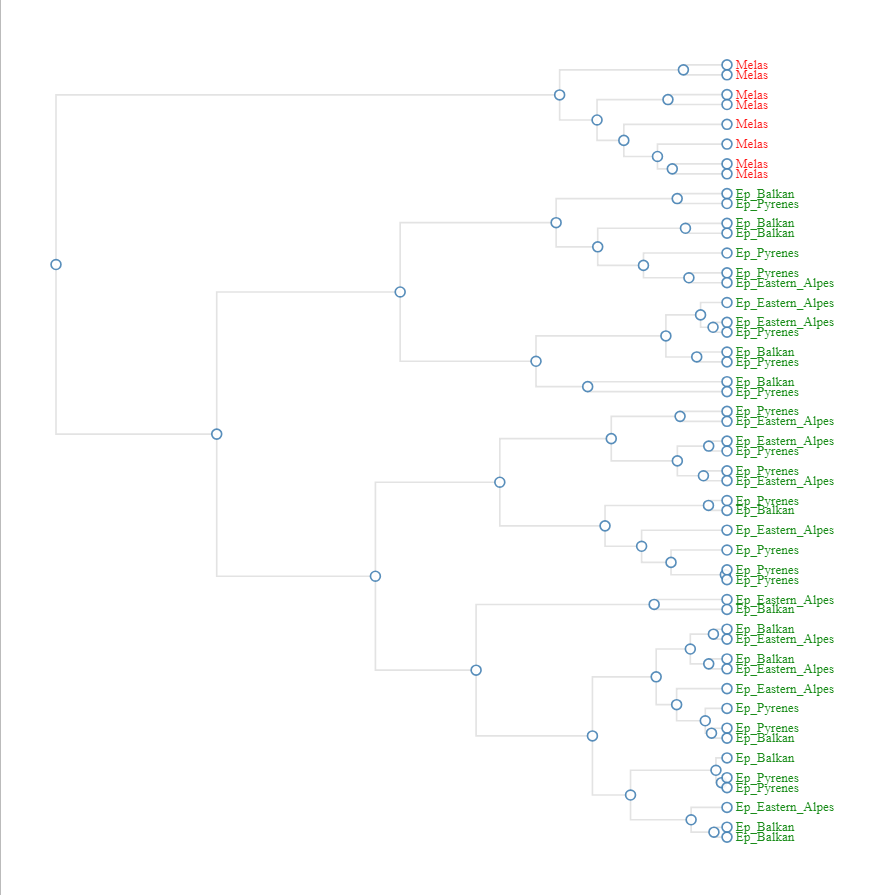


S11 Cluster Dendrogram (49 landmarks) of penis valves of *E. pronoe* and *E.melas* as reference species. Based on the euclydian distance.

S12 Principal component analysis with singular values and percentage explained by the relative warps.

| **PCs** | **SV** | **%** | **Cum %** | **PCs** | **SV** | **%** | **Cum %** |
| --- | --- | --- | --- | --- | --- | --- | --- |
| 1 | 0,64939 | 48,63% | 48,63% | 30 | 0,02264 | 0,06% | 99,67% |
| 2 | 0,42609 | 20,94% | 69,57% | 31 | 0,02092 | 0,05% | 99,72% |
| 3 | 0,28317 | 9,25% | 78,82% | 32 | 0,01928 | 0,04% | 99,76% |
| 4 | 0,19879 | 4,56% | 83,37% | 33 | 0,01779 | 0,04% | 99,80% |
| 5 | 0,15164 | 2,65% | 86,03% | 34 | 0,01663 | 0,03% | 99,83% |
| 6 | 0,14500 | 2,42% | 88,45% | 35 | 0,01544 | 0,03% | 99,86% |
| 7 | 0,13359 | 2,06% | 90,51% | 36 | 0,01489 | 0,03% | 99,88% |
| 8 | 0,12546 | 1,82% | 92,32% | 37 | 0,01264 | 0,02% | 99,90% |
| 9 | 0,10298 | 1,22% | 93,55% | 38 | 0,01231 | 0,02% | 99,92% |
| 10 | 0,08978 | 0,93% | 94,48% | 39 | 0,01186 | 0,02% | 99,94% |
| 11 | 0,08103 | 0,76% | 95,23% | 40 | 0,01122 | 0,01% | 99,95% |
| 12 | 0,07887 | 0,72% | 95,95% | 41 | 0,01013 | 0,01% | 99,96% |
| 13 | 0,07189 | 0,60% | 96,55% | 42 | 0,00904 | 0,01% | 99,97% |
| 14 | 0,06158 | 0,44% | 96,98% | 43 | 0,00820 | 0,01% | 99,98% |
| 15 | 0,05759 | 0,38% | 97,37% | 44 | 0,00727 | 0,01% | 99,98% |
| 16 | 0,05291 | 0,32% | 97,69% | 45 | 0,00598 | 0,00% | 99,99% |
| 17 | 0,04953 | 0,28% | 97,97% | 46 | 0,00578 | 0,00% | 99,99% |
| 18 | 0,04539 | 0,24% | 98,21% | 47 | 0,00535 | 0,00% | 100,00% |
| 19 | 0,04348 | 0,22% | 98,43% | 48 | 0,00443 | 0,00% | 100,00% |
| 20 | 0,04162 | 0,20% | 98,63% | 49 | 0,00359 | 0,00% | 100,00% |
| 21 | 0,03880 | 0,17% | 98,80% |  |  |  |  |
| 22 | 0,03793 | 0,17% | 98,97% |  |  |  |  |
| 23 | 0,03374 | 0,13% | 99,10% |  |  |  |  |
| 24 | 0,03098 | 0,11% | 99,21% |  |  |  |  |
| 25 | 0,03065 | 0,11% | 99,32% |  |  |  |  |
| 26 | 0,02650 | 0,08% | 99,40% |  |  |  |  |
| 27 | 0,02566 | 0,08% | 99,47% |  |  |  |  |
| 28 | 0,02503 | 0,07% | 99,55% |  |  |  |  |
| 29 | 0,02345 | 0,06% | 99,61% |  |  |  |  |


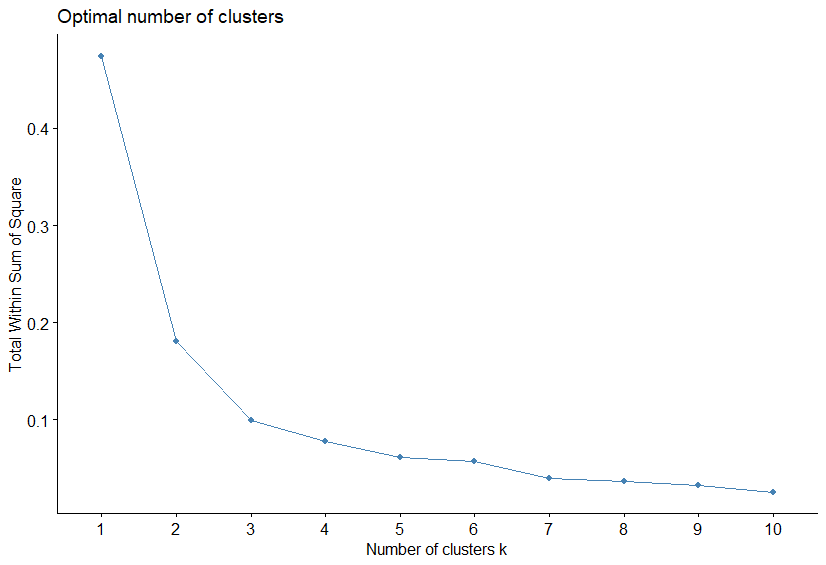


S13 Optimal number of cluster K for the morphology of penis valves of *E. pronoe* and *E.melas* as reference species.

S14 Relative contribution of each landmark of the valves of *E. pronoe*.

| **Landmark Nr.** | **SS** | **Landmark Nr.** | **SS** | **Landmark Nr.** | **SS** |
| --- | --- | --- | --- | --- | --- |
| 1 | 0,00087 | 17 | 0,02099 | 33 | 0,00022 |
| 2 | 0,00146 | 18 | 0,01329 | 34 | 0,00010 |
| 3 | 0,00067 | 19 | 0,00836 | 35 | 0,00007 |
| 4 | 0,00259 | 20 | 0,00246 | 36 | 0,00006 |
| 5 | 0,01421 | 21 | 0,00067 | 37 | 0,00005 |
| 6 | 0,03325 | 22 | 0,00067 | 38 | 0,00005 |
| 7 | 0,06644 | 23 | 0,00061 | 39 | 0,00004 |
| 8 | 0,13907 | 24 | 0,00051 | 40 | 0,00007 |
| 9 | 0,15045 | 25 | 0,00024 | 41 | 0,00014 |
| 10 | 0,12320 | 26 | 0,00051 | 42 | 0,00014 |
| 11 | 0,10489 | 27 | 0,00092 | 43 | 0,00011 |
| 12 | 0,08705 | 28 | 0,00055 | 44 | 0,00052 |
| 13 | 0,07672 | 29 | 0,00022 | 45 | 0,00139 |
| 14 | 0,06073 | 30 | 0,00019 |  |  |
| 15 | 0,04918 | 31 | 0,00020 |  |  |
| 16 | 0,03562 | 32 | 0,00023 |  |  |

S 15 Table 1 Geographical location of the analysed populations of *E. pronoe*

| **Population** | **Kürzel** | **East** | **North** |  |
| --- | --- | --- | --- | --- |
| Alisnica | Ali | 019.03630648 | 43.01950597 | 04.08.2014 |
| Babky | Bab | 019.25575314 | 49.06373272 | 01.08.2007 |
| Cauterets | Cau | 000.14444951 | 42.88263512 | 10.08.2013 |
| Col de Tourmalet | CT | 000.18655137 | 42.88357926 | 04.08.2013 |
| Etang de Areau | Etar | 001.13755862 | 42.73844451 | 26.07.2013 |
| GlocknerForschungsstation | GF | 012.59021205 | 46.87349964 | 14.08.2012 |
| Gletsch | Glet | 008.34917261 | 46.53948009 | 09.08.2005 |
| Grindelwald | Grin | 008.34917261 | 46.53948009 | 06.08.2003 |
| GrancharRila | GRR | 023.62406505 | 42.10001195 | 07.08.2011 |
| Hochkönig | HO | 013.03669011 | 47.44197926 | 13.08.2006 |
| Königsstein | KÖ | 025.25521250 | 45.54982944 | 13.08.2019 |
| Lac de Sayen | LDS | 000.22713762 | 42.94865481 | 09.08.2013 |
| Loser | LO | 013.73884252 | 47.60825146 | 05.08.2007 |
| Medvodje | Med | 014.71519500 | 46.41662963 | 18.07.2007 |
| Oisternig | OI | 013.49895954 | 46.49479040 | 05.08.2006 |
| Partnun | Pat | 009.83871852 | 46.96376659 | 07.08.2003 |
| Port de Laurrau | PDL | 000.99509131 | 42.92420538 | 12.08.2013 |
| Peca | Peca | 014.57227828 | 46.18152147 | 06.08.2006 |
| PassoGroce Domini | PGD | 010.37757524 | 45.77453490 | 12.08.2007 |
| Plöckenpass | Ploe | 012.95933898 | 46.58354109 | 04.08.2006 |
| PoscenskiKraj | Popkra | 019.08818954 | 42.99469424 | 02.08.2014 |
| Popova Shapka | Posha | 020.83895555 | 41.83845630 | 07-08.08.2011 |
| PassoPromollo | PPR | 013.27387304 | 46.54113982 | 17.08.2005 |
| Passo San Marco | PSM | 009.65906214 | 45.87224175 | 13.08.2007 |
| Seebergsattel | SE | 015.27724122 | 47.63096436 | 16.07.2007 |
| Simplonpass | SIM | 008.01663321 | 46.21699937 | 08.08.2005 |
| Vail d´Illiez | Vail | 006.89124460 | 46.16815996 | 08.08.2005 |

S16 GenBank accession numbers and corresponding haplotypes and genotypes.

| **mtDNA** | **CO1** | **ND1** | **Haplotypes** | **nDNA** | **RPS5** | **EF1alpha** | **Genotypes** |
| --- | --- | --- | --- | --- | --- | --- | --- |
| EP_Ali1 | MZ190632 | MZ345012 | H20 | EPALI1 | MZ190563 | MZ190678 | G54 |
| EP_Ali2 | MZ190633 | MZ345013 | H21 | EPALI2 | MZ190564 | MZ190679 | G64 |
| EP_Ali3 | MZ190634 | MZ345014 | H19 | EPALI3 | MZ190565 | MZ190680 | G53 |
| EP_Ali4 | MZ190635 | MZ345015 | H22 | EPCAU1 | MZ190566 | MZ190681 | G21 |
| EP_Bab1 | MZ190636 | MZ345016 | H39 | EPCAU2 | MZ190567 | MZ190682 | G20 |
| EP_CAU1 | MZ190637 | MZ345017 | H16 | EPCAU3 | MZ190568 | MZ190683 | G19 |
| EP_CAU2 | MZ190638 | MZ345018 | H12 | EPCAU5 | MZ190569 | MZ190684 | G9 |
| EP_CAU4 | MZ190639 | MZ345019 | H14 | EPCT1 | MZ190570 | MZ190685 | G8 |
| EP_CAU5 | MZ190640 | MZ345020 | H15 | EPCT2 | MZ190571 | MZ190686 | G4 |
| EP_CT1 | MZ190641 | MZ345021 | H10 | EPCT3 | MZ190572 | MZ190687 | G11 |
| EP_CT2 | MZ190642 | MZ345022 | H11 | EPCT4 | MZ190573 | MZ190688 | G2 |
| EP_CT4 | MZ190643 | MZ345023 | H9 | EPCT5 | MZ190574 | MZ190689 | G10 |
| EP_Etar1 | MZ190644 | MZ345024 | H6 | EPETAR1 | MZ190575 | MZ190690 | G7 |
| EP_Etar2 | MZ190645 | MZ345025 | H3 | EPETAR2 | MZ190576 | MZ190691 | G14 |
| EP_Etar3 | MZ190646 | MZ345026 | H7 | EPETAR3 | MZ190577 | MZ190692 | G3 |
| EP_KOE1 | MZ190647 | MZ345027 | H37 | EPETAR5 | MZ190578 | MZ190693 | G17 |
| EP_POPKRA3 | MZ190648 | MZ345028 | H18 | EPGF2 | MZ190579 | MZ190694 | G65 |
| EP_GF1 | MZ190649 | MZ345029 | H30 | EPGF3 | MZ190580 | MZ190695 | G62 |
| EP_Glet1 | MZ190650 | MZ345030 | H17 | EPGF4 | MZ190581 | MZ190696 | G66 |
| EP_Grin1 | MZ190651 | MZ345031 | H43 | EPGF5 | MZ190582 | MZ190697 | G61 |
| EP_GRR1 | MZ190652 | MZ345032 | H35 | EPGLET1 | MZ190583 | MZ190698 | G49 |
| EP_GRR2 | MZ190653 | MZ345033 | H41 | EPGLET2 | MZ190584 | MZ190699 | G48 |
| EP_HO1 | MZ190654 | MZ345034 | H36 | EPGLET3 | MZ190585 | MZ190700 | G47 |
| EP_HO3 | MZ190655 | MZ345035 | H40 | EPGRIN1 | MZ190586 | MZ190701 | G31 |
| EP_LDS1 | MZ190656 | MZ345036 | H13 | EPGRIN3 | MZ190587 | MZ190702 | G25 |
| EP_LDS3 | MZ190657 | MZ345037 | H8 | EPGRIN4 | MZ190588 | MZ190703 | G24 |
| EP_LO1 | MZ190658 | MZ345038 | H28 | EPGRIN5 | MZ190589 | MZ190704 | G30 |
| EP_LO4 | MZ190659 | MZ345039 | H38 | EPGRR3 | MZ190590 | MZ190705 | H58 |
| EP_Med1 | MZ190660 | MZ345040 | H24 | EPHO2 | MZ190591 | MZ190706 | G51 |
| EP_Pat2 | MZ190661 | MZ345041 | H44 | EPHO3 | MZ190592 | MZ190707 | G57 |
| EP_PDL1 | MZ190662 | MZ345042 | H4 | EPHO4 | MZ190593 | MZ190708 | G50 |
| EP_PDL2 | MZ190663 | MZ345043 | H2 | EPHO5 | MZ190594 | MZ190709 | G56 |
| EP_PDL3 | MZ190664 | MZ345044 | H5 | EPLDS1 | MZ190595 | MZ190710 | G6 |
| EP_PDL4 | MZ190665 | MZ345045 | H1 | EPLDS3 | MZ190596 | MZ190711 | G16 |
| EP_PECA1 | MZ190666 | MZ345046 | H23 | EPLDS4 | MZ190597 | MZ190712 | G18 |
| EP_PECA2 | MZ190667 | MZ345047 | H25 | EPLDS5 | MZ190598 | MZ190713 | G5 |
| EP_PGD1 | MZ190668 | MZ345048 | H29 | EPLO1 | MZ190599 | MZ190714 | G60 |
| EP_PGD2 | MZ190669 | MZ345049 | H27 | EPLO4 | MZ190600 | MZ190715 | G44 |
| EP_PLOE3 | MZ190670 | MZ345050 | H31 | EPMED3 | MZ190601 | MZ190716 | G67 |
| EP_PLOE5 | MZ190671 | MZ345051 | H32 | EPMED4 | MZ190602 | MZ190717 | G43 |
| EP_POSHA2 | MZ190672 | MZ345052 | H34 | EPOI1 | MZ190603 | MZ190718 | G23 |
| EP_PSM1 | MZ190673 | MZ345053 | H33 | EPPDL1 | MZ190604 | MZ190719 | G1 |
| EP_SE2 | MZ190674 | MZ345054 | H26 | EPPDL2 | MZ190605 | MZ190720 | G15 |
| EP_Vail1 | MZ190675 | MZ345055 | H42 | EPPDL4 | MZ190606 | MZ190721 | G13 |
| EP_Vail2 | MZ190676 | MZ345056 | H46 | EPPDL5 | MZ190607 | MZ190722 | G12 |
| EP_Vail5 | MZ190677 | MZ345057 | H45 | EPPGD1 | MZ190608 | MZ190723 | G40 |
|  |  |  |  | EPPGD2 | MZ190609 | MZ190724 | G39 |
| WSP1 | MZ358189 |  |  | EPPGD3 | MZ190610 | MZ190725 | G32 |
| WSP2 | MZ358190 |  |  | EPPGD5 | MZ190611 | MZ190726 | G38 |
| WSP3 | MZ358191 |  |  | EPPLOE1 | MZ190612 | MZ190727 | G63 |
|  |  |  |  | EPPLOE2 | MZ190613 | MZ190728 | G45 |
|  |  |  |  | EPPLOE3 | MZ190614 | MZ190729 | G46 |
|  |  |  |  | EPPLOE5 | MZ190615 | MZ190730 | G22 |
|  |  |  |  | EPPOSHA1 | MZ190616 | MZ190731 | G52 |
|  |  |  |  | EPPOSHA2 | MZ190617 | MZ190732 | G55 |
|  |  |  |  | EPPPR3 | MZ190618 | MZ190733 | G41 |
|  |  |  |  | EPPSM1 | MZ190619 | MZ190734 | G36 |
|  |  |  |  | EPPSM2 | MZ190620 | MZ190735 | G33 |
|  |  |  |  | EPPSM3 | MZ190621 | MZ190736 | G35 |
|  |  |  |  | EPPSM4 | MZ190622 | MZ190737 | G37 |
|  |  |  |  | EPPSM5 | MZ190623 | MZ190738 | G34 |
|  |  |  |  | EPSE3 | MZ190624 | MZ190739 | G59 |
|  |  |  |  | EPVAIL1 | MZ190625 | MZ190740 | G29 |
|  |  |  |  | EPVAIL2 | MZ190626 | MZ190741 | G28 |
|  |  |  |  | EPVAIL3 | MZ190627 | MZ190742 | G27 |
|  |  |  |  | EPVAIL5 | MZ190628 | MZ190743 | G26 |
|  |  |  |  | EPKOE1 | MZ190629 | MZ190744 | G68 |
|  |  |  |  | EPKOE3 | MZ190630 | MZ190745 | G42 |
|  |  |  |  | Erebiamelas1 | MZ190749 | MZ190747 | G69 |
|  |  |  |  | Erebiamelas6 | MZ190750 | MZ190748 | G70 |
|  |  |  |  | Erebiaaethiops | MZ190631 | MZ190746 |  |

1. Senckenberg Deutsches Entomologisches Institut, Systematik Und Biogeographie, Eberswalder Str. 90, 15374 Müncheberg, Germany [↑](#footnote-ref-2)
2. Universität Greifswald, Zoologisches Institut und Museum, Loitzer Straße 26, 17489 Greifswald, Germany [↑](#footnote-ref-3)
3. Department. Evolutionary Zoology, Faculty of Science and Technology, University of Debrecen, Egyetem-tér 1, Debrecen H-4010, Hungary [↑](#footnote-ref-4)
4. Department of Taxonomy and Ecology, Babes-Bolyai University RO-3400, Str. Clinicilor 5–7, Cluj-Napoca, Romania

   ^5^Entomology, Zoology, Institute of Biology, Faculty of Natural Sciences I, Martin Luther University Halle-Wittenberg, 06099 Halle (Saale), Germany

   ^6^Entomology and Biogeography, Institute of Biochemistry and Biology, Faculty of Science, University of Potsdam, 14476 Potsdam, Germany [↑](#footnote-ref-5)
